# Community-Acquired Urinary Tract Infection by* Escherichia coli* in the Era of Antibiotic Resistance

**DOI:** 10.1155/2018/7656752

**Published:** 2018-09-26

**Authors:** Dong Sup Lee, Seung-Ju Lee, Hyun-Sop Choe

**Affiliations:** Department of Urology, St. Vincent's Hospital, The Catholic University of Korea, College of Medicine, Republic of Korea

## Abstract

Urinary tract infections (UTIs) caused by* Escherichia coli (E. coli)* are the most common types of infections in women. The antibiotic resistance of* E. coli* is increasing rapidly, causing physicians to hesitate when selecting oral antibiotics. In this review, our objective is to ensure that clinicians understand the current seriousness of antibiotic-resistant* E. coli*, the mechanisms by which resistance is selected for, and methods that can be used to prevent antibiotic resistance.

## 1. Introduction

Community-acquired uncomplicated urinary tract infections (UTIs) account for a large proportion of infectious diseases in females [1], and a substantial amount of oral antibiotics is prescribed on a daily basis to treat UTIs among females in community-based outpatient clinics. Because* E. coli* accounts for up to 80% of community-acquired uncomplicated UTIs, these bacteria should be targeted when choosing empirical antibiotics [2]. In 2011, the Infectious Diseases Society of America (IDSA) recommended that trimethoprim-sulfamethoxazole (cotrimoxazole), nitrofurantoin, fosfomycin, or pivmecillinam be used if local resistance rates of uropathogens causing acute uncomplicated UTIs do not exceed 20% or if the infecting strain is known to be susceptible to these drugs [3]. Fluoroquinolones or beta-lactams such as cephalosporins are recommended as alternatives. Therefore, an awareness of regional susceptibility data regarding* E. coli* (antibiograms) is very important for selecting appropriate empirical antibiotics. However, the rate at which* E. coli* strains are becoming resistant to the vast majority of antibiotics is increasing worldwide. In addition,* Enterobacteriaceae* harbor gene(s) conferring resistance to almost all antibiotics [4] and plasmids harboring these resistance determinants can be transferred between bacteria, even between species, such that the acquisition of resistance to new antibiotics may only be a matter of time. Therefore, it is much more important to recognize practical rationales, including prescribing antibiotics when there is evidence of an infection, promoting appropriate use of antibiotics and increasing efforts for preventing UTIs. Following such strategies is essential because the abuse or misuse of antibiotics can lead to resistance via the emergence of mutant strains [5], and unresolved, relapsed UTIs tend to be resistant to previously used antibiotics [6].

Herein, we searched for an antibiotic resistance patterns for the past decade, especially with regard to oral antibiotics, reviewed the mechanisms of antibiotic resistance in* E. coli*, and suggest several strategies to overcome the challenges associated with these important issues.

## 2. Methods

We searched several databases, including PubMed, ISI Web of Science, Scopus, and Google Scholar using the following keywords: “*Escherichia coli*”* or *“*E. coli*”, “resistant” or “resistance”, “urinary tract infections” or “UTI”, “epidemiology”, “community”, and “acquired”. A key word was added when searching for regional antimicrobial susceptibilities, such as “Europe” or countries in Europe, such as “England”, “UK”, “France”, “Germany”, “Russia”, “Italy”, or “Spain”; “Asia” or countries in Asia, such as “China”, “Korea”, “Japan”, “Taiwan”, “Hong Kong”, “India”, or “Pakistan”; “America” or countries in North or South America, such as “US”, “Canada”, “Mexico”, or “Brazil”; “Mediterranean”, or countries in the Mediterranean region, such as “Greece” or “Turkey”; “Middle East” or countries in the Middle East region, such as “Egypt”, “Saudi Arabia”, or “Iran”; or “Australia”. When searching for the mechanisms of antibiotics resistance, “mechanism” was searched for with a key word, such as “co-trimoxazole” or “trimethoprim sulfamethoxazole”, “fluoroquinolone”, “beta-lactams” or “beta-lactamase”, “inhibitor”, “fosfomycin”, “nitrofurantoin”, or “carbapenemase”. When searching for antibiotic treatment or prevention, a word such as “strategy”, “treatment”, “management”, “preventive”, or “prevention” was added. During the review of articles, related articles on this subject were also reviewed.

## 3. Antimicrobial Susceptibility Pattern of* E. coli* in Community-Acquired Urinary Tract Infections for Oral Antibiotics in Recent Decades

There are limited oral options for the treatment of ESBL-producing bacteria associated with lower urinary tract infections (acute cystitis). Cotrimoxazole was a typical antibiotic used to treat UTIs, but the resistance of* E. coli* to this drug has markedly increased. According to the literature published in the past decade, in Asia, a 10~15% resistance rate to this drug was reported in Japan [7], with approximately 30% resistance rates observed in China and south Korea [8, 9]. In Europe and the Mediterranean region, the resistance rates of* E. coli* to cotrimoxazole varied but were usually over 15% [10–13]. However, there was an interesting report wherein the authors emphasized the role of cotrimoxazole in empirical antibiotics because of the recent decrease in the resistance rate to cotrimoxazole in several European countries due to its low prescription rate [14]. However, it may be not possible to reuse the drug worldwide within the next several years, and close observation of surveillance data will be required.

With respect to fluoroquinolones, in Japan and Australia, the susceptibility of* E. coli* to these drugs was approximately 90% [7, 15] and varied between 70~88% in the US [16] and 74~84% in China [8]. Middle and North European countries showed a fluoroquinolone susceptibility of 80% or greater [10, 11], while other European or some Mediterranean regions showed approximately a 60% susceptibility [12, 13]. Similar to cotrimoxazole, there was evidence that escape from exposure to this antibiotic will increase antimicrobial susceptibility in UTIs. According to Lee et al., the susceptibility of gram-negative bacteria to ciprofloxacin was much higher in patients less than 20 years old than in patients more than 20 years old. The reason for this observation may be the lower exposure to fluoroquinolones in young individuals because these drugs are not recommended for use in those under 20 years old [17].

One recent issue of importance is the increasing prevalence of extended spectrum beta lactamase- (ESBL-) producing* E. coli.* The prevalence of ESBL-producing* E. coli* has been increasing globally, as shown in Table 1 [12, 18–36]. Before 2010, the vast majority of countries showed less than a 5~10% prevalence of ESBL-producing* E. coli*, whereas the prevalence exceeded 10% in the local communities of many countries. Therefore, the increase in ESBL-producing* E. coli* is no different than that of cotrimoxazole-resistant* E. coli* or fluoroquinolone-resistant* E. coli*, and the prevalence of ESBL-producing* E. coli* is likely to increase soon.

Fosfomycin is an oral antibiotic agent that has broad activity against multidrug resistant (MDR), pathogens including ESBL-producing* E. coli*. Fosfomycin inhibits the synthesis of peptidoglycan at an earlier step than beta-lactam or glycopeptide antibiotics and has a broad spectrum of activity against various gram-positive and gram-negative bacteria, including methicillin-resistant* Staphylococcus aureus* and vancomycin-resistant* Enterococcus *spp. Fosfomycin has been shown to have advantages in the treatment of UTIs due to its high concentration in the urinary tract, which exceeds 2,000 mg/L after the initial administration and remains at high levels for a prolonged period, over 24 hours. However, fosfomycin should not be used for pyelonephritis or in patients with bacteremia due to inadequate concentrations within the bloodstream [37, 38]. Fosfomycin susceptibility in uropathogens, including* E. coli*, is currently greater than 90%, even in ESBL-producing* E. coli* [39–41].

Another oral antimicrobial agent that can be considered for the treatment of ESBL-producing* E. coli* cystitis is nitrofurantoin. Nitrofurantoin is a drug that has been used since 1950s to treat uncomplicated UTIs and works by damaging bacterial DNA in its highly active reduced form. Now, and even in earlier eras of widespread use, the baseline resistance to nitrofurantoin was low (0–5%) [42, 43]. Nitrofurantoin should only be used for lower UTIs, and its use should be avoided in patients with a creatinine clearance of less than 60 mL/minute, as reduced renal function results in decreased active drug within the urine [44].

**Figure 1:**
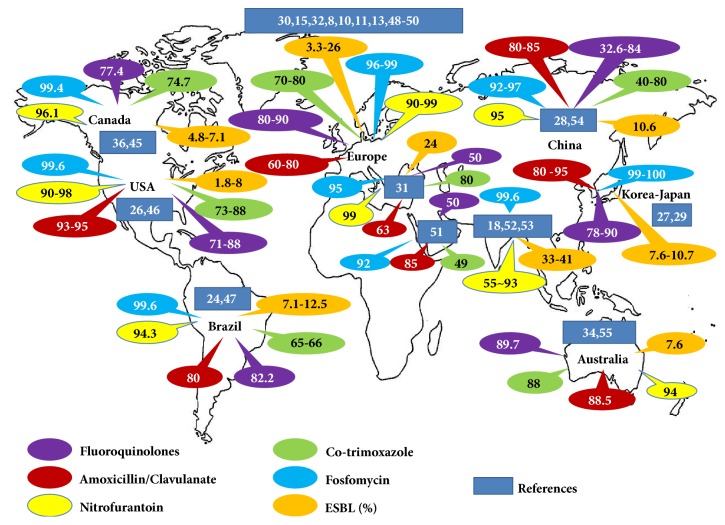
Worldwide susceptibilities of* E. coli* to oral antibiotics in community-acquired urinary tract infections in the last decade.

Figure 1 [7–13, 15, 18, 20, 21, 23, 27, 33, 35, 36, 45–55] shows the efficacies of several oral antibiotics against* E. coli* in community-acquired uncomplicated UTIs. Because fosfomycin and nitrofurantoin have not been included in the antimicrobial formularies of many institutes, it is difficult to achieve previous susceptibility data. Furthermore, because of the history of disappointing in vitro results at the beginning of fosfomycin-susceptibility testing, the use of the drug has been limited in the US and in many other countries [56]. Interestingly, these older medicines have become more important because of the high sensitivity of* E. coli* to these drugs in the era of antibiotic resistance. Recent studies have shown that these antibiotics have over 90~95% efficacies in almost all areas studied (Figure 1), although there may be no way to predict another decrease in the use of these drugs for UTI treatment.

## 4. Plasmid-Mediated Dissemination of Antibiotic Resistance Determinants

Plasmid is a generic term for DNA molecules other than chromosomes that can independently replicate in bacterial cells [57]. Although not necessary under most circumstances, plasmids can encode genes that promote bacterial survival and can be transferred to their descendants. Antimicrobial resistance determinants are some of the most important elements carried by plasmids. Although plasmids can be transmitted to other species via conjugation without becoming integrated into DNA [58], sometimes plasmids can integrate into chromosomal DNA for replication. Types of plasmids associated with ESBL-producing* E. coli* include IncFII, IncN, and IncI1, among which IncI1 resistance plasmids are known to contribute to CTX-M type ESBL dissemination in* E. coli* [59]. Resistance determinants against most antimicrobial agents can be conferred from species to species via plasmids, which is why systematic monitoring for antibiotic resistance in communities is important, as is the education of staff concerning infection control and prevention, especially in intensive care units. The next section will describe the details of epidemiological studies of plasmid-mediated resistance in* E. coli* infections.

## 5. Mechanisms of Action and Resistance to Anti-*E. coli* Drugs and Its Microbiological Epidemiology

Because tetrahydrofolate is required to make both purines and pyrimidines, its synthesis is important for understanding the mechanism of cotrimoxazole, which is a combination of trimethoprim and sulfamethoxazole. Trimethoprim is a structural analog of dihydrofolic acid that competitively inhibits the synthesis of tetrahydrofolic acid. Sulfamethoxazole, which has a sulfonyl group instead of a carbonyl group, is an analog of para-aminobenzoic acid that competitively inhibits the synthesis of dihydrofolic acid. Over two decades after its first use in 1974 [60], this drug has remained the first-line treatment for uncomplicated UTIs in adults [61]. Because of the widespread resistance to the drug, cotrimoxazole has been gradually replaced by fluoroquinolones since approximately the year 2000 [62]. The mechanism of bacterial resistance to cotrimoxazole is due to (1) drug efflux pumps, (2) the degradation of the antibiotics by enzymes, (3) the alteration of antibiotic binding targets, and (4) the loss of drug entry points, all of which can occur via chromosomal mutations or the acquisition of plasmids [63].

Fluoroquinolones have a keto acetic acid group where fluoroquinolone−topoisomerase binding is facilitated through a water−metal ion bridge [64]. Eventually, fluoroquinolone−topoisomerase complex inhibits topoisomerase activity, and subsequently DNA replication is blocked. Acquisition of resistance to fluoroquinolones is from both chromosome and plasmid. Chromosomal-mediated resistance decrease in fluoroquinolone uptake and the expression of efflux pumps. Plasmid-encoded proteins which are associated with fluoroquinolones resistance are (1) Qnr proteins which decrease topoisomerase-DNA binding and protects enzyme-DNA complexes from quinolones, (2) Aac(6′)-lb-cr which acetylate the free nitrogen of the C7 ring of the quinolones, and (3) plasmid-encoded efflux pumps such as QepA1 and QepA2 [64, 65].

As shown in Figure 1, *β*-lactams, which are typically prescribed clinically in medical offices, are losing their efficacy in many areas because of the loss of their activity against* E. coli*. The mechanism of resistance of* E. coli* to *β*-lactams is essentially due to plasmid-mediated transmission of genes encoding *β*-lactamases. In contrast, in* Klebsiella *species, another species that causes uncomplicated urinary tract infection encodes a *β*-lactamase (e.g., SHV) on its chromosome [66]. It is not easy for general physicians to understand the classification of *β*-lactamases. In brief, according to the preferences of researchers, two classifications have been used to describe *β*-lactamase, including molecular classification from A to D and functional classification with regard to activity against *β*-lactamase inhibitors [67]. In general, class C corresponds to functional group 1, classes A and D correspond to group 2, and class B corresponds to group 3. ESBLs comprise group 2be (molecular class A) and can hydrolyze penicillin and first generation cephalosporins but can be inhibited by clavulanic acid and tazobactam. ESBLs can hydrolyze at least one of the following antibiotics at a 10% increased rate over that of benzylpenicillin: cefotaxime, ceftazidime, and/or aztreonam [67, 68]. TEM, a common genotype of *β*-lactamase, is named by a patient whose name was Temoneira, although the origin of TEM has not been identified precisely [69]. TEM-1 and TEM-2 belong to the group 2b (class A) *β*-lactamase genotype and have the ability to hydrolyze penicillin and/or first-generation cephalosporin, whereas CTX-M-15, which has recently become the most well-known genotype among *β*-lactamases, is common in ESBL-producing* E. coli* and is affiliated with group 2be (class A). The origin of CTX-M *β*-lactamases is believed to be from chromosomal ESBL genes in* Kluyvera* spp. [70]. A large-scale investigation (72 hospitals) in the US conducted in 2012 revealed that CTX-M-15 was the most common genotype in ESBL-producing* E. coli* [71]. Meanwhile, many recent studies have emphasized that the relative proportion of sequence type 131* E. coli* (*E. coli* ST131) is predominant among all ESBL-producing* E. coli* [72, 73] and have shown that CTX-M-15 was encoded in plasmids and was transferred horizontally [74, 75]. When we compared research on non-ESBL-producing and fluoroquinolone-resistant ST131* E. coli* isolates [76] to another study in which ST131* E. coli* produced CTX-M-15 beta-lactamase and was resistant to fluoroquinolone [77], it appears that CTX-M-15 may be a *β*-lactamase that is acquired from plasmids in fluoroquinolone-resistant* E. coli* [78]. AmpC-type *β*-lactamases (group 1, class C) are encoded in chromosomes and are inducible by antibiotic pressure, such as amoxicillin. Beta-lactam antibiotics such as cefoxitin induce AmpC expression by binding to transpeptidases (penicillin-binding proteins), which results in a balance shift to murein degradation that subsequently activates the transcriptional regulator AmpR and increases its promoter activity [79]. These *β*-lactamases are generally resistant to clavulanic acid, cephalosporins, and cephamycin and can be expressed by* Citrobacter *spp.*, Serratia *spp., and* Enterobacter *spp. but are rarely observed in* E. coli* [80, 81]. Dissemination of AmpC can occur via the mobilization of chromosomal AmpC genes from different enteric bacteria, such as C. freundii and E. cloacae, and their subsequent horizontal transfer to other species [82]. The first plasmid-borne AmpC gene identified was CMY-1 [83], followed by MIR-1 and CMY-2. Currently, CMY-2-type genes have been suggested to be one of the most common plasmid-borne AmpC enzymes [84]. According to a previous study by Sidjabat et al., a single IncI1 plasmid carrying* bla*CMY-2 was predominant among different clones of* E. coli*, suggesting the occurrence of horizontal transfer of this IncI1,* bla*CMY-2-carrying plasmid [85].

OXA family *β*-lactamases (group 2d, class D) hydrolyze oxacillin at a faster rate (> 50%) than that observed for benzylpenicillin. OXA-related *β*-lactamases have recently been identified in plasmids from* E. coli* [86] that exhibit low-level resistance to imipenem and resistance to ertapenem. Plasmid-mediated dissemination of OXA-48-like carbapenemases in* E. coli* has been observed in many European countries [87].

Besides OXA family *β*-lactamases,* K. pneumoniae* carbapenemase (KPC: group 2f, Class A) and metallo-beta-lactamases (MLBs: group 3, class B) are important types of carbapenemases. KPC enzyme in clinical isolate was first identified in 1996 [88]. In recent decade, the KPC determinants are identified world-widely and the incidence rate is rapidly increasing. KPC producers have been reported mostly from hospital-acquired* K. pneumoniae* isolates, but KPC-producing* E. coli* and other enterobacterial species have also been described [89]. In a report from Italian nationwide surveillance from outpatients, 93.2% of carbapenemases were associated with* bla*KPC type carbapenemase where the majority came from* K. pneumoniae* and 4.2% from* E. coli* [90]. Especially with respect to* E. coli*, Kalyan et al. emphasized that spread of* bla*KPC producing* E. coli* is mainly caused by horizontal transfer of* bla*KPC harboring plasmids such as IncFIA, IncFII_K1_, IncFII_K2_, or IncN [91].

Although MBLs were originally known as chromosomally encoded enzymes, the most frequently identified MBLs (IMPs and VIMs) have been observed to be encoded in plasmids from the family* Enterobacteriaceae* [92]. MBLs of the IMP-type appear to be primarily restricted to the Asian continent and are only rarely identified in Europe among enterobacterial isolates. In contrast, VIM-type MBLs have been identified worldwide in enterobacterial isolates responsible for large hospital outbreaks [93, 94]. NDM-1 (New Delhi metallo-ß-lactamase) is highly prevalent in the Indian subcontinent but has also been identified in many countries worldwide, demonstrating its rapid dissemination. The* bla*NDM-1 gene is primarily plasmid-associated.

Fosfomycin, which was discovered in 1969 [95], inhibits bacterial wall (peptidoglycan) biosynthesis by acting as an analog of phosphoenolpyruvate and binding UDP-GlcNAc enopyruvyl transferase, inactivating the enzyme [96]. Fosfomycin resistance has been identified in some bacteria that resulted from the mutation of UDP-GlcNAc enopyruvyl transferase [97]. However, in* E. coli*, both mutation-induced resistance and acquired resistance can occur. Several fosfomycin modifying enzymes, including FosA, encoded by plasmid-borne genes can confer fosfomycin resistance in* E. coli*.

Nitrofurantoin is one of the few drugs that can be used during pregnancy [98]. By oxygen-insensitive nitrofuran reductase, active intermediates of the drug can transferred into the bacteria where they act upon ribosimes and DNA [99], although the precise mechanisms of action of these intermediates have not yet been identified. The nfsA and nfsB genes, encoding nitroreductases, are encoded in* E. coli* [100]. Mutation in these genes in bacteria can lead to resistance to this drug. According to recent report, bacterial resistance to nitrofurantoin was shown be mediated by OqxAB efflux pumps [101], and the authors described that mutation in the resistant determinant genes nfsA and nfsB could be transmitted by plasmid.

Colistin is sensitive to most of gram negative bacteria, even to* bla*NDM-1 producing* Enterobacteriaceae* [102]. The action of colistin is known that cationic structure of the drug binds with anionic lipopolysaccharides causing displacement of cationic (calcium and magnesium) peptides from the outer cell membrane of gram negative bacteria, leading to disruption of the outer membrane and permeability change [103]. The mechanism of resistance has not been clearly understood, but several mechanisms such as outer membrane modification, over-expression of efflux pump, and overproduction of capsule polysaccharide have been suggested [104]. Recent data shows that plasmid mediated gene such as MCR-1 plays a great role on horizontal dissemination of colistin resistance in community [105]. In addition, Mao et al. emphasized that exposure to antibiotics elicited the emergence of MDR* E. coli* harboring MCR-1 [106].

## 6. Risk Factors for Acquisition of Antimicrobial Resistance in* E. coli*

Antibiotic exposure is the most important factor for the selection of antimicrobial resistance. Lee et al. described that increased exposure to fluoroquinolones/cephalosporins made bacteria more resistant to fluoroquinolone/cephalosporins [30]. Although it is not fully understood in detail how antibiotic resistance arises in microorganisms after their exposure to antibiotics, Baquero suggested that exposure to very low antibiotic concentrations can select for low-level resistant mutants, which serve as stepping stones to the strains with high-level resistance [5]. Similarly, Cantón et al. suggested that the use of an antibiotic at a concentration capable of preventing the generation of mutants, above the minimal inhibitory concentration, would restrict the emergence of such first-step mutants within a susceptible population [107]. Undesirable exposure to antibiotics typically occurs due to the abuse or misuse of antibiotics. In many countries, antibiotics can be obtained over the counter and are as easy to obtain as aspirin and cough medicine [108], which is a major contributing factor to antibiotics abuse. Recently, a study from India, where resistant rate of antibiotics has been relatively high, investigated antibiotics misuse where participants with limited access to an allopathic doctor, either for logistical or economic reasons, were observed to be more likely to purchase medications directly from a pharmacy without a prescription [109]. In the United States 20 years ago, experts estimated that at least half of the human therapeutic use of antibiotics in the United States was unnecessary or inappropriate [4].

Colonization has also been suggested to be risk factor for the selection of antimicrobial resistance. Most clinical factors associated with colonization and infection by ESBL-producing organisms involve healthcare exposure, such as hospitalization, residence in a long-term care facility, hemodialysis use, and the presence of an intravascular catheter [110, 111]. In a study of Dutch individuals who had no ESBL colonization prior to international travel, 34 percent overall and 75 percent of individuals who travelled to southern Asia became colonized by ESBL-producing strains following their travels [112]. Another report showed an ESBL prevalence of 49.0-64.0% for residents and 5.2-14.5% for staff [113]. Thus, travelers to endemic areas, hospitalized patients, care-givers in health care unit, guardians of in-patients, and hospital workers, including residents, are at an increased risk of colonization by antimicrobial resistant bacteria, showing the importance of environmental hygiene and taking precautions against contact with MDR bacteria. Once a cluster of resistant bacteria colonizes any part of the human body, it is possible that the bacteria will grow and horizontally transfer plasmid-encoded resistance genes to other susceptible bacteria or to different species [58].

Another important route of slow encroachment by resistant bacteria is the dispensing of antibiotics into ecosystems. Harrison et al. demonstrated that human ingestion of animal and plant food products carries a strong potential for the spread of antibiotic resistance genes via the consumption of antibiotic residues and antibiotic-resistant bacteria [4]. The authors concluded that the continued use of antibiotics in livestock and other agricultural endeavors may soon make these drugs ineffective for human therapeutic use.

Finally, indwelling catheters, which lead to complicated UTIs, are a known a risk factor for the acquisition of MDR bacteria [114].

## 7. Complicated UTIs and MDR Gram-Negative Bacteria

Classically, UTIs with functional or anatomical abnormalities of the urinary tract are named complicated UTIs [115]. With respect to complicated UTIs, treatment of asymptomatic bacteriuria has not been shown to be beneficial; it could increase the risk of the development of antimicrobial-resistant uropathogens [116]. Antimicrobial resistance is more common in complicated UTIs [42], which may be because patients with complications are more vulnerable to UTIs and are more likely to be exposed to antibiotics, catheterization, and hospital sources.

Meanwhile, a major point in complicated UTIs is the concept of drainage of infected materials to reduce treatment periods and prevent infections from ascending to upper tract. Lee et al. emphasized that drainage of prostatic abscesses would reduce the period of antibiotic administration [117]. A report of long-term bladder management in spinal cord injury showed that condom catheter use (passive drainage) increases the vulnerability of patients to severe infection rather than intermittent catheterization (active drainage) [118].

Catheter-associated UTIs have multiple confounding factors associated with emerging antimicrobial resistance, that is, hospital factors due to frequent hospitalization, foreign body bridges between the urinary bladder and the outside of the body, and frequent antibiotic exposure. A previous study conducted with outpatient UTIs showed that catheter-associated UTIs were more closely associated to exposure to antibiotics and exhibited a higher occurrence of infection caused by an atypical organism, such as* Citrobacter *species,* Proteus mirabilis*,* Morganella morganii*,* Enterobacter species*, and* Pseudomonas aeruginosa* rather than* E. coli* [17]. Those atypical organisms can harbor MDR determinant and can transfer the resistance determinants (e.g., AmpC gene) to* E. coli *(see Section 5). Therefore, regarding plasmid-mediated resistance determinants, catheter associated UTIs obviously contribute to the emergence of resistant strains. Therefore, in managing neurogenic bladder with high bladder volume or high bladder pressure, avoidance of unnecessary catheterization and/or a change in the catheterization method used, from indwelling catheters to clean intermittent catheterization, must be considered [42].

A recent study recommended the use of amoxicillin/clavulanate (or amoxicillin plus aminoglycoside), cefixime, ceftibuten, levofloxacin, ciprofloxacin, and fosfomycin as empirical antibiotics against catheter-associated UTIs, whereas recommended regimens for empiric treatment of uncomplicated UTIs were fosfomycin, nitrofurantoin and pivmecillinam [119]. However, cultivation should be performed prior to the use of empirical antibiotics, especially in complicated UTIs because atypical and/or MDR microorganisms are more likely to be isolated.

## 8. Antibiotic Treatment of UTIs by MDR Gram-Negative Bacteria

In regard to laboratory cut-off values of microbial load (10^3^ cfu/ml, 10^4^ cfu/ml, or 10^5^ cfu/ml), it is very difficult to determine treatment initiation in certain cut-off value. However, bacterial count is usually 10^2^ ~ 10^4^ cfu/ml in many patients with UTIs, and half of women with symptomatic cystitis have bacteriuria lower than 10^5^ cfu/ml [120]. Franz et al. also suggested antibiotic treatment in symptomatic patients with microbial load between 10^2^ and 10^5^ cfu/ml [121]. Furthermore, when severe infection such as urinary sepsis was suspected, physicians should start antibiotic therapy before the cultivation report (no time to wait laboratory microbial count) because of its high mortality. Therefore, symptoms (or signs) may be more important factor than the cut-off values of laboratory microbial load to initiate the treatment of UTIs.

Clinical studies have indicated that delayed treatment with inappropriate antibiotics for MDR bacteremia would exert a negative influence on patient mortality [122, 123]. Therefore, when a MDR bacterial infection is suspected and previous cultivation reports are not available, or when a patient has a systemic illness, physicians may be obligated to choose parenteral agents such as piperacillin/tazobactam or carbapenems empirically. In these circumstances, patients require hospitalization.

Currently, the emergence of many types of carbapenemases has caused a sense of crisis for physicians. However, several new drugs have been developed that have allowed physicians to save the use of carbapenems. Ceftazidime/avibactam is a new cephalosporin *β*-lactamase inhibitor combination targeting to* Enterobacteriaceae* and* Pseudomonas aeruginosa*, which can be used as an alternative to carbapenems for infections caused by ESBL- or AmpC-producing gram-negative bacteria [124, 125]. Ceftolozane/tazobactam can also be used as an alternative to carbapenems to treat ESBL-producing gram negative infections [126].

A recent study suggested a treatment algorithm for MDR gram-negative bacterial infections [127]. If an MDR bacterial infection is suspected, and there was no past carbapenem resistance, it is recommended to use parenteral amoxicillin/clavulanate or piperacillin/tazobactam followed by oral fosfomycin, nitrofurantoin or pivmecillinam plus amoxicillin/clavulanate, unless patients have systemic illness, whereas if there was no susceptibility data or patients have systemic illness, it is recommended to use carbapenems, temocillin, or ceftolozane/tazobactam. If carbapenem resistance had been noted, specific antibiotics should be considered according to the local policy of avoiding the development of antibiotic resistance. Therefore, investigations concerning the types of carbapenemases and their surveillance are very important. For example, if a cultivation assay result identifies a strain as being resistant to carbapenemase and metallo-*β*-lactamases is known as a prevalent type in the local area, physicians can use colistin or tigecycline [128].

## 9. Contradiction between the Use of and Resistance of Antibiotics, Focusing Acute Cystitis

The fact that exposure to antibiotics increases resistance is a problem for clinicians who need to continue to treat infected patients. It is true that the use of antibiotics should be reduced to decrease the development of strains that are resistant to antibiotics. However, excessive limitation of antibiotics for treating symptomatic UTI or for prophylaxis may lead to another cost increase due to recurrence. Therefore, we need to develop a strategy to adequately control urinary tract infections while minimizing the increase in antibiotic resistance.

First, a management strategy should be developed for systemic and localized infections. In the case of pyelonephritis that causes systemic infections, including UTI sepsis, broad spectrum antibiotics should be used intensively. When a course of antibiotics is started empirically, the choice of agent should be reevaluated once culture results are available. Continuous surveillance of antibiotic resistance patterns by region is essential for the appropriate selection of antibiotics for empirical treatment.

Reducing the total amount of antibiotic use is important for resistance control. The use of cephalosporins and quinolones and the long-term use of antibiotics have been identified as risk factors for infections caused by extended-spectrum ESBL* E. coli* and* Klebsiella *species [129, 130]. The use of broad spectrum antibiotics to treat systemic infection may be inevitable. However, total antibiotic usage is higher in non-febrile uncomplicated UTIs (in other words, acute cystitis). Finally, an effective strategy for controlling antibiotic resistance is to use antibiotics appropriately for cystitis treatment and to prevent recurrent cystitis using available means.

Furthermore, this strategy is more important and necessary because of the occurrence of collateral damage, which describes increased colonization or infection by MDR organisms with the use of broad-spectrum antimicrobials, including fluoroquinolones and cephalosporins [131, 132]. Thus, fluoroquinolones and cephalosporins are no longer recommended as the first-line treatment for acute uncomplicated cystitis in the EAU and IDSA guidelines. Instead, drugs that cause minimal resistance and have a propensity for collateral damage are recommended as the first-line treatment, such as nitrofurantoin, fosfomycin, and pivmecillinam [3, 133].

Obviously, the best way to treat UTIs should be to use optimal antibiotics instead of empirical treatment, if possible, based on culture findings [134]. This principle is the same whether it is simple cystitis or febrile UTI. Of course, it is well known that worldwide socio-economic status varies from region to region, and a full laboratory examination cannot be performed for all patients [135, 136]. Therefore, it is not possible to implement consistent guidelines with respect to laboratory diagnostics. However, we should be aware of the high incidence of infectious diseases, even if they cause low mobility or mortality, such as cystitis. It is also important to strengthen the natural defense mechanism of the human body for helping recovery from the infected condition and to prevent recurrence of the same disease, rather than blind antibiotic prescription.

## 10. Prevention of Infectious Disease Is the Best Option for Antibiotic Resistance Control, Especially in Recurrent UTI

Recurrent UTI is defined as recurrence of uncomplicated and/or complicated UTIs, with a frequency of at least three UTIs/year or two UTIs in the last six months. Generally, UTIs differ from other infectious diseases in which pathognomonic sources are transmitted from the outside, such as sexually transmitted infections or respiratory tract infections. The bacteria that cause UTIs have characteristics that are typically symbiotic with the human body, and when an imbalance arises for some reason, it can result in infections of the urinary tract [137]. Even individuals who do not have a urinary system abnormality are at risk to become infected, and some individuals suffer from repeated UTIs without apparent cause. Moreover, a person with a structural or functional abnormality of the urinary tract is at a higher risk for urinary tract infections. For this reason, efforts to manage the exposure of humans to infectious agents can reduce the incidence of urinary tract infections, and these efforts may help to slow the development of antibiotic resistance [58].

## 11. Proper Bladder Emptying

All physicians treating UTIs should have awareness of urogenital anomalies and the need for proper bladder emptying. Congenital urinary tract abnormalities should be investigated for pediatric UTIs. For adults, close history taking can identify functional factors, such as bladder outlet obstruction or underactive bladder caused by spinal cord lesions. Physicians should also pay attention to noticeable problems, such as spinal cord injury and congenital anomalies in the urogenital system, as well as inconspicuous problems, such as BPH or DM polyneuropathy. Urogenital imaging studies are performed when evaluating recurrent UTIs, but additional measurements such as postvoid residual urine volume should be considered [138, 139]. If there is a problem in bladder function, proper bladder emptying methods, such as clean intermittent catheterization (CIC), the use of urethral catheters, or the administration of some medications, such as alpha-blockers for sphincter relaxation, should be actively explored. Proper emptying of the bladder is the most important factor in recurrent UTI control.

## 12. Nonantimicrobial Prophylaxis

The active use of nonantimicrobial prophylaxis is often indicated and does not result in an increase in antimicrobial resistance of the commensal flora, as nonantimicrobial prophylaxis, immunoactive agents, probiotics (*Lactobacillus *spp.), cranberry-based products, D-mannose, hormonal replacement (in postmenopausal women), and others have been studied [140–144]. Among these modalities, the urinary immunopotentiator is now well documented and strongly recommended in the guidelines [145]. The oral immunostimulant OM-89 (Uro-Vaxom®), an extract of 18 different serotypes of heat-killed uropathogenic* E. coli*, stimulates innate immunity by increasing non-specific and specific humoral and cellular immune responses by stimulating the production of interferon-*γ* and tumor necrosis factor-*γ*, as well as the activities of lymphocytes and macrophages [146–148]. Uro-Vaxom® is a safe and effective medicine that can reduce recurrent UTI episodes [140, 149–151] and can effectively reduce the repeated use of antibiotics [152]. Physicians need to actively use immunoactive agents that have been proven effective rather than letting patients find a solution or take medication on their own. However, a weakness of these agents is that they cannot completely prevent the recurrence of an infection and require relatively long-term use. Therefore, to increase compliance, it is important to provide sufficient information to the patient and to establish a good doctor-patient relationship.

## 13. Awareness of Asymptomatic Bacteriuria (ABU)

ABU should be distinguished from symptomatic UTI. ABU occurs in an estimated 1-5% of healthy pre-menopausal females, increasing to 4-19% in otherwise healthy elderly females and men, also occurring in 0.7-27% of patients with diabetes, 2-10% of pregnant women, 15-50% of institutionalized elderly patients, and 23-89% of patients with spinal cord injuries [153, 154]. ABU does not cause systemic influences, such as renal damage [155]. Thus, treatment of ABU is not recommended in patients without risk factors [153]. Furthermore, ABU should not be overtreated without the awareness of the physician [156]. Even in catheterized patients (also see Section 7), antibiotics should be considered only when patients with indwelling catheters present symptoms or they have any complications during placement or exchanges of catheters [157]. In contrast, considering ABU in pregnancy, many researches recommended treating the UTIs because not to treat UTIs in pregnant women can increase the possibilities of preterm labor or low birth-weight [158].

The major risk factor for ABU is diabetes mellitus. DM, even when well regulated, is reported to correlate to a higher frequency of ABU [159]. Considering the high morbidity and mortality of symptomatic UTI in DM, patients should be sufficiently treated by nonantibiotic methods. In particular, bladder dysfunction, such as diabetic cytopathy, should be carefully considered, and proper bladder emptying must be encouraged with diverse methods [160]. Even in these cases, immunopotentiators can be a good option for preventing symptomatic UTIs.

## 14. Pain Control for Cystitis Patients

For patients with nonfebrile uncomplicated cystitis, active pain control, and minimal use of antibiotics should be prioritized. Uncomplicated cystitis can be a self-limited disease in many cases. One study even showed that only symptomatic care using NSAID could be as effective as antibiotics in acute cystitis [161]. Pain in acute cystitis is a natural consequence of the inflammatory response, and pain-mediated urinary frequency or urgency is the chief complaint of patients. Therefore, for this self-limited disease, pain killers, including NSAIDs, may be a good option for symptomatic care as well as reducing the consumption of antibiotics. Delayed treatment is also a good strategy for antimicrobial-sparing [162]. Another caution is the abuse or misuse of overactive bladder (OAB) medicine with anticholinergic effects for acute urgency or urge incontinence in UTIs. Overuse of anticholinergic medicine can interfere with proper bladder emptying, and adverse effects with respect to UTI control may occur.

## 15. Antimicrobial Stewardship

Antibiotics are overused across the world through their prescription, self-medication, or over-the-counter (OTC) availability. With the quantity of antibiotic use linked to antibiotic resistance, society should seek to preserve the use of this irreplaceable resource through education and regulation [163].

Antimicrobial stewardship programs aim to optimize the outcomes of prevention and treatment of infection while curbing the overuse and misuse of antimicrobial agents [145, 164, 165]. Antimicrobial stewardship has a positive clinical impact on UTIs caused by ESBL-producing* E. coli* [166]. To this end, antimicrobial therapy should be tailored to each patient, taking into consideration the severity of disease, individual and local patterns of antimicrobial resistance and the potential for collateral damage associated with antimicrobial use. Selecting the correct drug, dose, as well the shortest clinically effective duration of therapy when possible, is key to optimal antimicrobial stewardship [134]. Some prescription strategies should be considered carefully, including the following [167]:

- Precise indication for antibiotic treatment
- Choice of the appropriate compound
- Appropriate dosage
- Adequate route of administration
- Administration timing and treatment length

All physicians who treat UTIs should take on the responsibility of antimicrobial stewardship.

## 16. Conclusion

Even if new antibiotics are introduced and appear on the market, the development of resistance to these antibiotics by* E. coli* will begin immediately. The mechanisms by which* E. coli* becomes resistant to antibiotics vary greatly with the antibiotic, but genes conferring resistance can be transmitted via plasmids among species. Therefore, the implementation of antibiotic stewardship programs is crucial to minimize the chance of selecting for resistant resistance. Such programs should be founded on the following principles: (1) antibiotics should be used when there is evidence of a bacterial infection to minimize the unnecessary exposure of patients to antibiotics; (2) ABU should not be treated (if there is no risk factor) to minimize unnecessary exposure to antibiotics; (3) cultivation before using antibiotics and using appropriate antibiotics (if possible, considering using nitrofurantoin, fosfomycin, or pivmecillinam as first-line antibiotics) should be performed according to regional susceptibility data to decrease the chance of “collateral damage”; (4) the use of appropriate antibiotic doses, not underdoses, to potentially reduce mutant formation; and (5) the use of antibiotics for appropriate durations to reduce recurrence (appropriate de-escalation with repeated culture). In addition, to prevent overuse of antibiotics, self-medication, or over-the-counter (OTC) availability should be limited by education or regulation. Furthermore, paying attention to hygiene, especially individuals who travel to endemic areas or who are frequently in circumstances with a high risk of exposure to antibiotic resistant bacteria (e.g., healthcare units), could reduce the chance of colonization by resistant micro-organisms. Finally, the use of nonantimicrobial prophylaxis could effectively reduce the total amount of antibiotic consumption.

In general, cotrimoxazole has under 80% sensitivity and fluoroquinolones have approximately 80% sensitivity, but the latter drug shows under 60% sensitivity in some parts of Asia, the Middle-East, and the Mediterranean region. Beta-lactam with inhibitor, amoxicillin/clavulanate, shows approximately 80% sensitivity, except for some European countries and the Mediterranean region. South Asia is an endemic region of ESBL producing* E. coli*. Yet, nitrofurantoin and fosfomycin show over 90% of sensitivity in the most of countries of the world.

## Figures and Tables

**Table 1:** The prevalence of extended spectrum beta lactamase-producing *E. coli* in community acquired urinary tract infections before and after 2010.

	Before 2010	After 2010	References
Europe			
UK^a^	4.6%	6.6%	[18]
France	1.1%	3.3%	[19, 20]
Spain	2.4~18.2%	8.9~23.6%	[21–23]
Mediterranean region			
Italy	3.5%	6.7%	[24, 25]
Turkey	8~13.1%	24%	[12, 26]
South Asia	21.7%	33.2%	[27]
Far east Asia	4.8~7.5%^b^	7.6~10.7%	[28–30]
Latin America	1.7%^c^	7.1~12.5%	[31–33]
US and Canada	7.4	1.8~8%	[34–36]

a: Possibly contaminated by a nosocomial source.

b: The source of the specimens maybe from community acquired UTIs but was not described precisely.

c: The data were collected from multinational sources.
